# A redox cycle with complex II prioritizes sulfide quinone oxidoreductase-dependent H_2_S oxidation

**DOI:** 10.1016/j.jbc.2021.101435

**Published:** 2021-11-19

**Authors:** Roshan Kumar, Aaron P. Landry, Arkajit Guha, Victor Vitvitsky, Ho Joon Lee, Keisuke Seike, Pavan Reddy, Costas A. Lyssiotis, Ruma Banerjee

**Affiliations:** 1 Department of Biological Chemistry, University of Michigan Medical School, Ann Arbor, Michigan, USA; 2 Department of Molecular and Integrative Physiology, Michigan Medicine, University of Michigan, Ann Arbor, Michigan, USA; 3 Department of Internal Medicine, Michigan Medicine, University of Michigan, Ann Arbor, Michigan, USA; 4 University of Michigan Medical School, Ann Arbor, Michigan, USA

**Keywords:** hydrogen sulfide, complex II, electron transport chain, coenzyme Q, fumarate, SDHA, CoQ, coenzyme Q, DES, diethyl succinate, DMF, dimethyl fumarate, DMI, dimethyl itaconate, DMM, dimethyl malonate, ETC, electron transport chain, *nd*SQOR, nanodisc-embedded SQOR, NNT, nicotinamide nucleotide transhydrogenase, OCR, oxygen consumption rate, PNC, purine nucleotide cycle, ROS, reactive oxygen species, SQOR, sulfide quinone oxidoreductase

## Abstract

The dual roles of H_2_S as an endogenously synthesized respiratory substrate and as a toxin raise questions as to how it is cleared when the electron transport chain is inhibited. Sulfide quinone oxidoreductase (SQOR) catalyzes the first step in the mitochondrial H_2_S oxidation pathway, using CoQ as an electron acceptor, and connects to the electron transport chain at the level of complex III. We have discovered that at high H_2_S concentrations, which are known to inhibit complex IV, a new redox cycle is established between SQOR and complex II, operating in reverse. Under these conditions, the purine nucleotide cycle and the malate aspartate shuttle furnish fumarate, which supports complex II reversal and leads to succinate accumulation. Complex II knockdown in colonocytes decreases the efficiency of H_2_S clearance while targeted knockout of complex II in intestinal epithelial cells significantly decreases the levels of thiosulfate, a biomarker of H_2_S oxidation, to approximately one-third of the values seen in serum and urine samples from control mice. These data establish the physiological relevance of this newly discovered redox circuitry between SQOR and complex II for prioritizing H_2_S oxidation and reveal the quantitatively significant contribution of intestinal epithelial cells to systemic H_2_S metabolism.

The discovery of H_2_S as an endogenously synthesized signaling molecule in mammals has fueled a growing literature on its physiological effects (1). Mechanistic insights into how H_2_S modulates cellular responses are, however, scarce (2, 3), and much attention has been focused on protein persulfidation, a reactive posttranslational modification of cysteine (4) that has been identified in hundreds of proteins (5, 6). On the other hand, the best characterized cellular effects of H_2_S are its oxidation *via* a dedicated mitochondrial pathway (7) or by globins (8, 9, 10) and its inhibition of complex IV (11) in the electron transport chain (ETC), leading to respiratory poisoning (Fig. 1*A*). The mitochondrial sulfide oxidation pathway begins with the conversion of H_2_S to glutathione persulfide catalyzed by sulfide quinone oxidoreductase (SQOR), an inner mitochondrial membrane flavoprotein (12). Electrons released from H_2_S oxidation are transferred to coenzyme Q (CoQ) and enter the ETC at the level of complex III, making H_2_S an inorganic substrate for oxidative phosphorylation in mammals (13). The remainder of the pathway successively converts glutathione persulfide to thiosulfate and, in some cells, to sulfate (14). The role in signaling, if any, of the reactive sulfur species formed during H_2_S oxidation remains to be fully elucidated (15). In this study, we report that a noncanonical redox circuit is established when complex IV is inhibited, *via* reversal of complex II activity to prioritize H_2_S oxidation.

**Figure 1:**
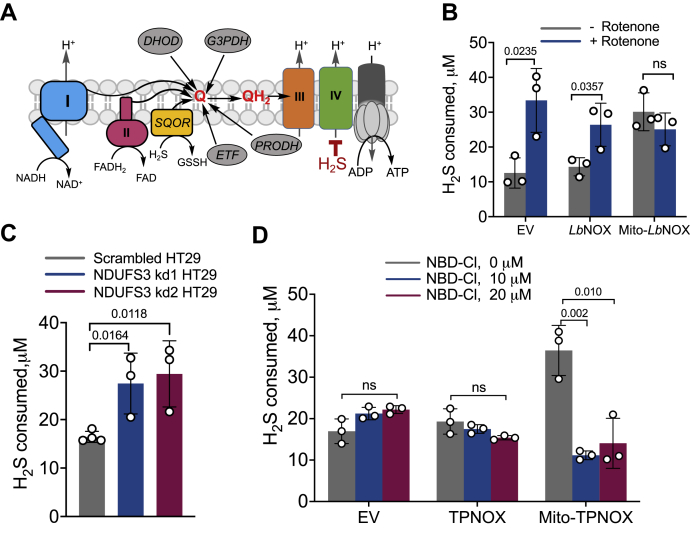
**The mitochondrial NADH pool influences the efficiency of H**_**2**_**S oxidation in HT29 cells.***A*, scheme showing that multiple CoQ (Q) users compete with SQOR including complexes I and II, dihydroorotate dehydrogenase (DHOD), glycerol 3-phosphate dehydrogenase (G3PDH), proline dehydrogenase (PRODH), and the electron transfer flavoprotein (ETF). *B*, H_2_S oxidation is enhanced in cells expressing mitochondrial but not cytoplasmic *Lb*NOX *versus* the empty vector (EV) control. Rotenone (2 μM) enhanced H_2_S clearance in control and cytoplasmic but not mitochondrial expressing *Lb*NOX cells. *C*, disruption of complex I by NDUFS3 knockdown enhanced H_2_S oxidation. *D*, mitochondrial expression of TPNOX accelerates H_2_S oxidation, which is inhibited by NBD-Cl. The data represent the mean ± S.D. of three independent experiments. ns, not significant.

SQOR functions as a respiratory shield, sensitizing the ETC to H_2_S poisoning when its activity is attenuated (16). At low H_2_S concentrations, however, SQOR activity increases respiration as measured by the oxygen consumption rate (OCR) (17). The dual potential to stimulate electron flux and inhibit the ETC raises questions as to whether modulation of mitochondrial bioenergetics by H_2_S is pertinent to its cellular signaling mechanism and fans out to other compartments *via* redox and metabolomic changes (2).

SQOR is one of several consumers of CoQ (Fig. 1*A*), and sulfide oxidation is impaired in CoQ deficiency (18). SQOR activity has the potential to cause a reductive shift in the CoQ pool, particularly at H_2_S concentrations that partially or fully inhibit complex IV. H_2_S also indirectly perturbs the NAD^+^/NADH and FAD/FADH_2_ couples that are connected to CoQ/CoQH_2_ *via* the ETC. We have previously demonstrated that H_2_S induces a reductive shift in the NAD^+^/NADH redox couple, creating an electron acceptor insufficiency that leads to uridine and aspartate deficiency and enhanced reductive carboxylation (16). While uridine limitation results from the CoQ dependence of dihydroorotate dehydrogenase in the pyrimidine pathway (Fig. 1*A*), aspartate deficiency results in part from reduced flux through the TCA cycle and the NADH-linked malate-aspartate shuttle. Furthermore, H_2_S stimulates the Warburg effect, enhancing glucose consumption and lactate production (19), and stimulates lipid biogenesis (20).

The effects of H_2_S on the ETC itself have received scant attention (13, 19, 21). The observed increase in succinate and decrease in malate at H_2_S concentrations that inhibit respiration were proposed to result from complex II reversal (13). While the same authors later proposed that H_2_S induces reverse electron transfer through complex I (17), neither model was evaluated experimentally. A recent study on oligomycin-treated murine microglia reported increased OCR upon exposure to an H_2_S donor and interpreted this as evidence of reverse electron transfer through complex I (22). The known drivers of mitochondrial reverse electron transfer, which leads to reactive oxygen species (ROS) generation, are a high membrane potential and an overreduced CoQ pool (23). Since respiratory poisons depolarize the mitochondrial inner membrane by limiting electron-coupled proton transfer (Fig. 1*A*), the premise for H_2_S-induced reverse electron transfer is unclear. Furthermore, the study contradicted the reported lack of H_2_S-induced ROS production (24).

Studies in our laboratory have focused primarily on colonic epithelial cells (16, 19, 20) that are routinely exposed to high concentrations of H_2_S from gut microbiota, estimated to range from ∼0.2 to 2.4 mM (25, 26). In this study, we report that rewiring within the ETC circuitry *via* complex II reversal prioritizes H_2_S oxidation under conditions of respiratory poisoning with fumarate serving as an electron acceptor. These results have important implications for understanding the mechanism by which intestinal epithelial cells respond to routine exposure to high H_2_S levels derived from the microbiota and potentially, the role of H_2_S in signaling a shift in energy metabolism.

## Results

### SQOR catalyzes sulfide-dependent reduction of O_2_

We examined whether O_2_ can serve an alternate electron acceptor for SQOR since complex IV poisoning by H_2_S should not restrict O_2_ availability (Fig. S1*A*). We found that when nanodisc-embedded SQOR (*nd*SQOR) (27) was reduced in the presence of sulfide and sulfite but in the absence of CoQ, O_2_ consumption was stimulated (Fig. S1*B*). From the linear dependence of OCR on O_2_ concentration, a *k*_on_ of 3370 ± 290 M^−1^ s^−1^ was estimated (Fig. S1*C*). Oxygen (*k* ∼ 14 min^−1^ at 75 μM O_2_) is, however, a significantly less efficient electron acceptor than CoQ (15 × 10^3^ min^−1^ at 75 μM CoQ) (27).

In the presence of a slight excess of sulfide (10 μM) and sulfite (15 μM), SQOR (7.5 μM) catalyzed the consumption of an equimolar concentration of O_2_ (7.3 ± 0.6 μM) (Fig. S1*D*). This reaction stoichiometry predicted that the products of O_2_ reduction by SQOR could be either O_2_^•−^ and FADH• or H_2_O_2_ and FAD. The equivalence between the O_2_ consumed and the concentration of H_2_O_2_ produced (7.6 ± 0.6 μM) is consistent with the two-electron reduction of O_2_ by SQOR (Fig. S1*A*). The concentration of H_2_O_2_ was significantly diminished (0.2 ± 0.1 μM) when catalase was added to the reaction mixture. The approximately 1:1:1 stoichiometry of sulfide added:O_2_ consumed:H_2_O_2_ produced is consistent with electron transfer from FADH_2_ to O_2_ *via* a C4a-hydroperoxy FAD intermediate (Fig. S1*E*), as proposed in other O_2_-activating flavoenzymes (28).

### Complex I activity decreases the efficiency of H_2_S oxidation

Complex I-dependent oxidation of NADH with concomitant reduction of CoQ is a major source of electron flux in the ETC and is expected to influence the efficiency of H_2_S oxidation. We have previously reported that H_2_S causes a reductive shift in the NAD^+^/NADH ratio by inhibiting complex IV (16). H_2_S oxidation was unaffected by the cytoplasmic, but significantly enhanced by the mitochondrial expression of the water forming NADH oxidase, *Lb*NOX (29) (Fig. 1*B*). Rotenone, a complex I inhibitor, increased H_2_S oxidation in control and *Lb*NOX but not mito-*Lb*NOX cells (Fig. 1*B*). Knockdown of NDUFS3 (Fig. S2), which is required for complex I assembly, increased H_2_S oxidation (Fig. 1*C*). Collectively, these data demonstrate that the cellular H_2_S oxidation capacity can be limited by the mitochondrial NADH pool.

The mitochondrial NADH and NADPH pools are interconnected *via* the activity of the electrogenic nicotinamide nucleotide transhydrogenase (NNT) located in the inner mitochondrial membrane. Cytoplasmic expression of TPNOX, a genetically encoded water forming NADPH oxidase (30), had no effect on H_2_S oxidation, while mitochondrial expression enhanced clearance (Fig. 1*D*). The NNT inhibitor NBD-Cl (4-chloro-7-nitrobenzofurazan chloride) attenuated the mito-TPNOX effect, further demonstrating that the capacity for cellular H_2_S oxidation is linked to the status of the mitochondrial NAD(P)H redox pool (Fig. 1*D*).

### Succinate accumulates in response to H_2_S

**Figure 2:**
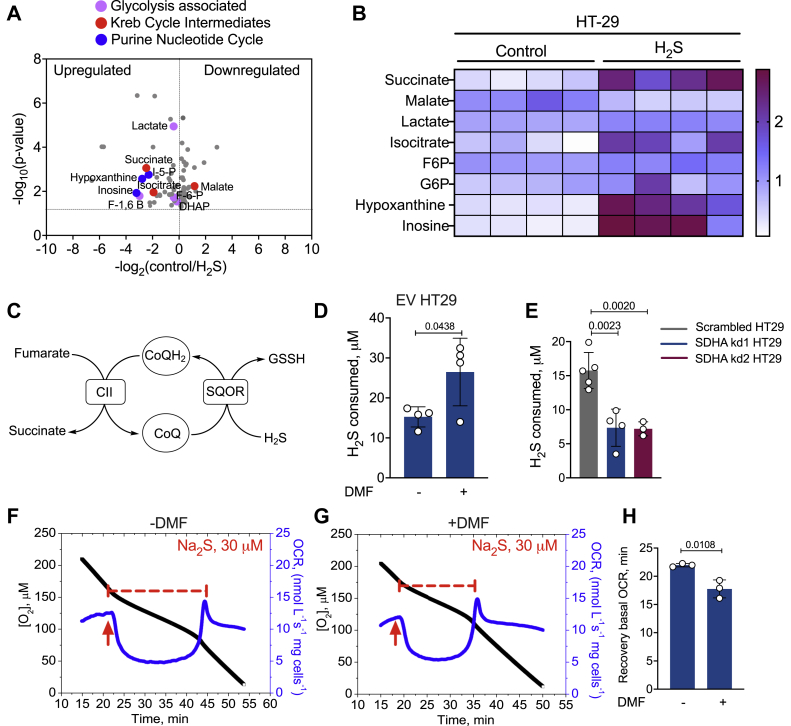
**H**_**2**_**S induces succinate accumulation through reversal of complex II activity.***A*, volcano plot showing changes in select metabolite in response to Na_2_S (100 μM) treatment of HT29 cells for 1 h and represent a replot of the metabolomics data reported in (16). The *gray* and *colored dots* represent metabolites that exhibit statistically significant perturbations in response to H_2_S treatment. A subset of the metabolites that are pertinent to this study are highlighted in *blue*, *pink*, and *red* as noted. *B*, heat map showing H_2_S-induced changes in select metabolites observed in four independent technical repeats. *C*, scheme showing how complex II reversal can regenerate CoQ for H_2_S oxidation. *D*, DMF (100 μM) increases H_2_S oxidation in EV HT29 cells. *E*, SDHA knockdown in HT29 cells reduces H_2_S oxidation. *F* and *G*, the duration of respiratory inhibition in HT29 cells by H_2_S is longer in the absence (*F*) *versus* presence (*G*) of DMF (200 μM). The *red arrows* indicate when H_2_S (30 μM) was added. *H*, comparison of the time required by HT29 cells to return to the basal respiration rate ± DMF. The data in (*D* and *E*) represent the mean ± S.D. of 3 to 4 independent experiments.

Metabolomics analysis after exposure to Na_2_S (100 μM, 1 h) revealed a number of changes in glycolytic, TCA cycle (16), and purine metabolism intermediates in malignant HT29 cells (Fig. 2, *A* and *B*). Interestingly, H_2_S treatment led to ∼5.5-fold higher levels of succinate. To test whether succinate accumulation resulted from reversal of complex II activity (Fig. 2*C*), we used dimethyl fumarate (DMF), a membrane permeable derivative of fumarate that increases intracellular fumarate concentration (31). DMF accelerated H_2_S oxidation in four out of five colorectal carcinoma lines but not in RKO cells (Figs. 2*D* and S3). The molecular basis of the difference in response between RKO and the other cell lines is presently unclear. Two other complex II inhibitors, dimethyl malonate and dimethyl itaconate, also inhibited H_2_S clearance, while diethyl succinate did not (Fig. S4). Knocking down SDHA (Fig. S5), the complex II subunit that catalyzes the reversible oxidation of succinate to fumarate, reduced H_2_S clearance (Fig. 2*E*). DMF shortened the recovery time for return to basal OCR following respiratory inhibition by H_2_S in HT29 (Fig. 2, *F*–*H*), HCT116, LoVo, and DLD cells (Fig. S6) but had no effect when SDHA was knocked down in HT29 cells (Fig. S7). Together, these data are consistent with the model that H_2_S oxidation is facilitated by reversal of complex II activity.

### The effect of complexes I and II on H_2_S-dependent OCR

**Figure 3:**
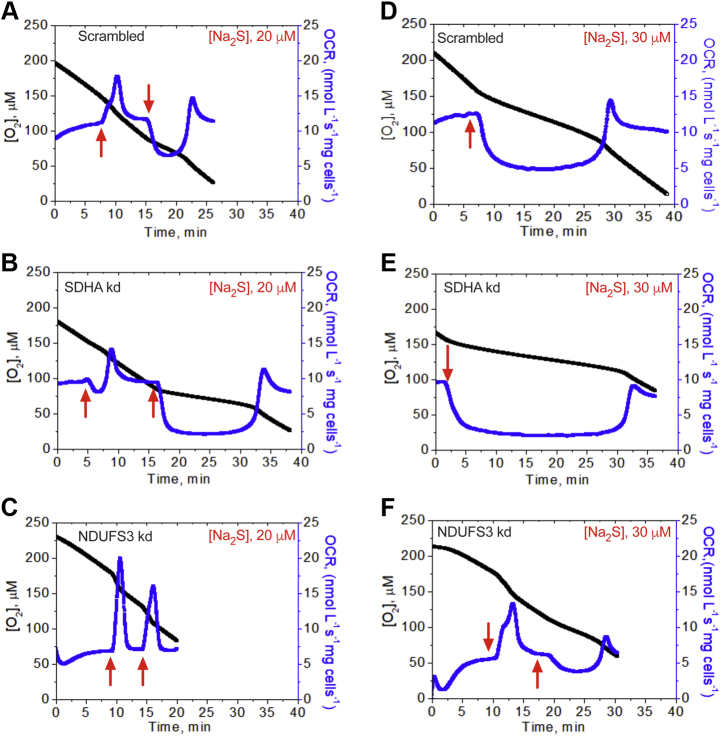
**Complexes I and II influence H**_**2**_**S-linked OCR.** Comparison of OCR activation with H_2_S (20 or 30 μM) in (*A* and *D*) scrambled, (*B* and *E*) SDHA knockdown, and (*C* and *F*) NDUFS3 knockdown HT29 cells. *Red arrows* indicate when H_2_S was added. The traces are representative of 3 to 5 independent experiments.

To further test the influence of complexes I and II on the cellular response to H_2_S, OCR was monitored in control *versus* NDUFS3 and SDHA knockdown cells. NDUFS3 knockdown decreased basal OCR twofold (Fig. 3), consistent with complex I being a major entry point for electrons into the ETC. At a low concentration of H_2_S (10 μM), OCR activation in NDUFS3 knockdown cells was robust, and the peak increase in OCR was higher than in control and SDHA knockdown cells (Fig. S8). At a higher H_2_S (20 μM) concentration, differences between the cell lines were clearly visible (Fig. 3, *A*–*C*). While the NDUFS3 knockdown showed robust activation of OCR in response to H_2_S, the control and SDHA knockdown cells showed signs of inhibition. The SDHA knockdown cells also took a longer time to recover basal OCR compared with controls. Following the first and second 20 μM H_2_S injection, control and SDHA knockdown cells showed signs of partial and severe respiratory inhibition, respectively, in contrast to NDUFS3 knockdown cells. At a higher H_2_S concentration (30 μM), control and SDHA knockdown cells responded with net inhibition of oxygen consumption in comparison to NDUFS3 knockdown cells, which exhibited a mixed response (Fig. 3, *D*–*F*). These results indicate that the CoQ pool limits sulfide clearance and, in the absence of competition from complex I, cells clear sulfide more efficiently. The data also reveal that complex II has the opposite effect, *i.e.*, it is advantageous for sulfide clearance, consistent with our model that complex II reversal supports H_2_S oxidation by catalyzing CoQH_2_ oxidation.

### Malate-aspartate shuttle and PNC furnish fumarate in H_2_S treated cells

**Figure 4:**
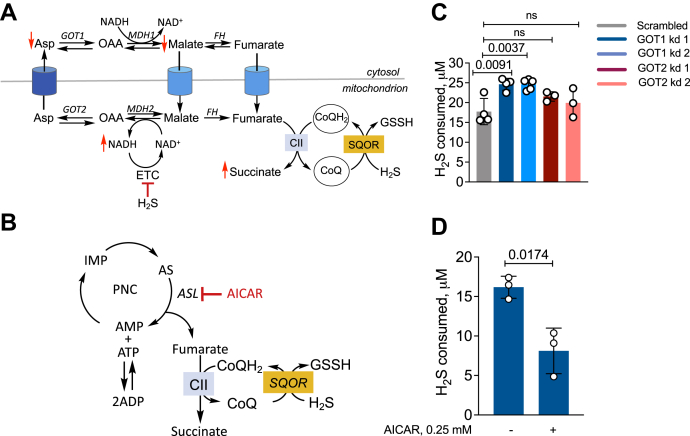
**The PNC and the malate-aspartate shuttle support fumarate-driven H**_**2**_**S oxidation.***A* and *B*, schemes showing that the malate-aspartate shuttle (*A*) and the PNC (*B*) are fumarate sources and that AICAR inhibits adenylosuccinate lyase (ASL). MDH1/2, FH, OAA, and CII denote malate dehydrogenase 1/2, oxaloacetate, fumarate hydratase, and complex II, respectively. *C*, H_2_S oxidation is stimulated in GOT1 knockdown but unaffected by GOT2 knockdown in HT29 cells. *D*, AICAR (0.25 mM) inhibits H_2_S clearance. The data in (*C* and *D*) represent the mean ± S.D. of 3 to 4 independent experiments. ns, not significant.

Since the malate-aspartate shuttle and the purine nucleotide cycle (PNC) (Fig. 4, *A* and *B*) are metabolic sources of fumarate in ischemic cells (23), we tested whether they also contribute to fumarate when the ETC is inhibited by H_2_S. For this, GOT1 and GOT2 (glutamic-oxaloacetic aminotransferases 1 and 2) expressed in the cytoplasm and mitochondrion, respectively, were knocked down in HT29 cells (Fig. S9). GOT1 but not GOT2 knockdown increased H_2_S oxidation by ∼38% compared with control cells (Fig. 4*C*). GOT1 knockdown also promoted H_2_S clearance as reflected by the shorter recovery time to the basal respiration rate (Fig. S10). Inhibition of adenylosuccinate lyase with AICAR (5-aminoimidazole-4-carboxamide ribonucleotide) decreased H_2_S clearance by ∼50% (Fig. 4*D*), consistent with a role for the PNC in this process.

### SDHA knockout in murine intestinal epithelial cells decreases H_2_S oxidation

**Figure 5:**
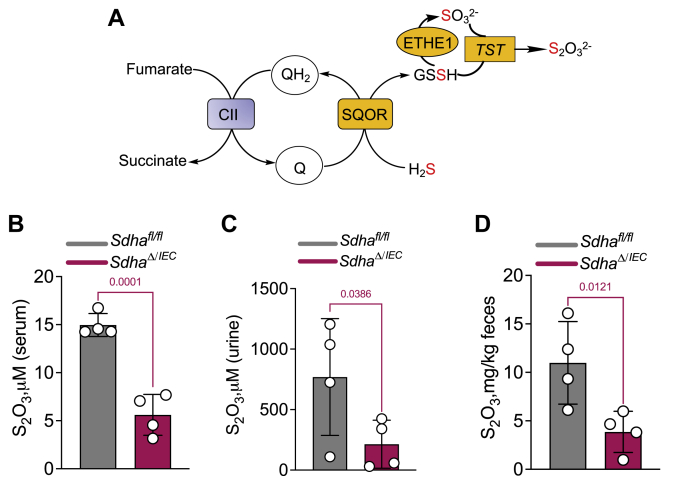
**Villin**^**Cre**^**SDHA**^**fl/fl**^**mice have reduced thiosulfate levels.***A*, scheme connecting H_2_S oxidation to thiosulfate production. *B*–*D*, quantitation of thiosulfate levels in control (*Sdha*^fl/fl^) and *Villin*^*Cre*^*Sdha*^fl/fl^ knockout mice in serum (*B*), urine (*C*), and feces (*D*). The data represent the mean ± S.D. for samples collected from four mice in each group.

To assess the physiological relevance of our observation that H_2_S clearance is supported by complex II working in reverse, we measured the impact of attenuating complex II on organismal H_2_S metabolism. For this, mice harboring loxP-flanked *Sdha* were crossed to mice expressing Cre recombinase under control of the villin promoter to specifically target intestinal epithelial cells, to generate *Vil1-Cre Sdha*^fl/fl^ (*Sdha*^*ΔIEC*^) mice as described previously (32). The rationale for targeting intestinal epithelial cells is that they are routinely exposed to high concentrations of H_2_S (25, 26) and actively oxidize sulfide (16). Thiosulfate, a stable product of H_2_S oxidation (Fig. 5*A*), is a handy biomarker of H_2_S metabolism (19). H_2_S, on the other hand, is difficult to measure due to its volatility and low steady-state concentrations in biological samples (33). *Sdha*^*ΔIEC*^ mice showed significantly lower thiosulfate levels compared with control *Sdha*^fl/fl^ (Fig. 5, *B*–*D*) revealing that the loss of complex II in intestinal cells caused local (feces) and systemic (serum and urine) perturbations in H_2_S oxidation.

## Discussion

In this study, we have uncovered a new mechanism for clearing H_2_S when its concentrations rise to levels that inhibit complex IV and preclude the use of O_2_ as the terminal electron acceptor for SQOR-dependent H_2_S oxidation. Such conditions might be relevant in the gut epithelium (where H_2_S exposure is high) or in ischemia (where O_2_ supply is cut off). Reversal of complex II activity under such conditions supports SQOR-dependent H_2_S oxidation, using fumarate as an alternate electron acceptor and prioritizes H_2_S clearance.

Metabolomic changes in HT29 cells in response to H_2_S provided clues to reprogramming driven changes that could potentially impact its clearance. Hypoxanthine and succinate, classic ischemic biomarkers (23, 34), also accumulate in response to H_2_S (Fig. 2*B*). Ischemic succinate accumulation is derived from oxidative TCA cycle metabolism (35) as well as from complex II-catalyzed reduction of fumarate (23). Fumarate is derived *via* the malate-aspartate shuttle and the PNC (23). Since H_2_S decreases the NAD^+^/NADH ratio and stimulates reductive carboxylation of α-ketoglutarate (16), the effect of the oxidative TCA cycle on H_2_S clearance was not examined. The PNC and the malate aspartate shuttle both impacted H_2_S clearance (Fig. 4, *C* and *D*). The PNC is activated in response to a drop in the adenylate energy charge (36) and is consistent with lower ATP levels in H_2_S-treated cells (19) as well as the observed increase in inosine, which is formed *via* deamination of adenosine.

Knockdown of GOT1, but not GOT2, increased the efficiency of H_2_S clearance, suggesting that the cytoplasmic arm of the malate-aspartate shuttle is an important source of fumarate. H_2_S leads to aspartate deficiency (16), potentially stimulating GOT1-catalyzed transamination of oxaloacetate to aspartate rather than the reverse, which is consistent with lower malate levels in H_2_S-treated cells (Fig. 2*B*). In GOT1 knockdown cells, oxaloacetate should be more available for malate dehydrogenase catalyzed reduction to malate, which can be dehydrated to fumarate (Fig. 4*A*) by fumarate hydratase that is present in the cytoplasm and the mitochondrion (37). Cytosolic fumarate can potentially enter the mitochondrion *via* a dicarboxylate carrier (38).

**Figure 6:**
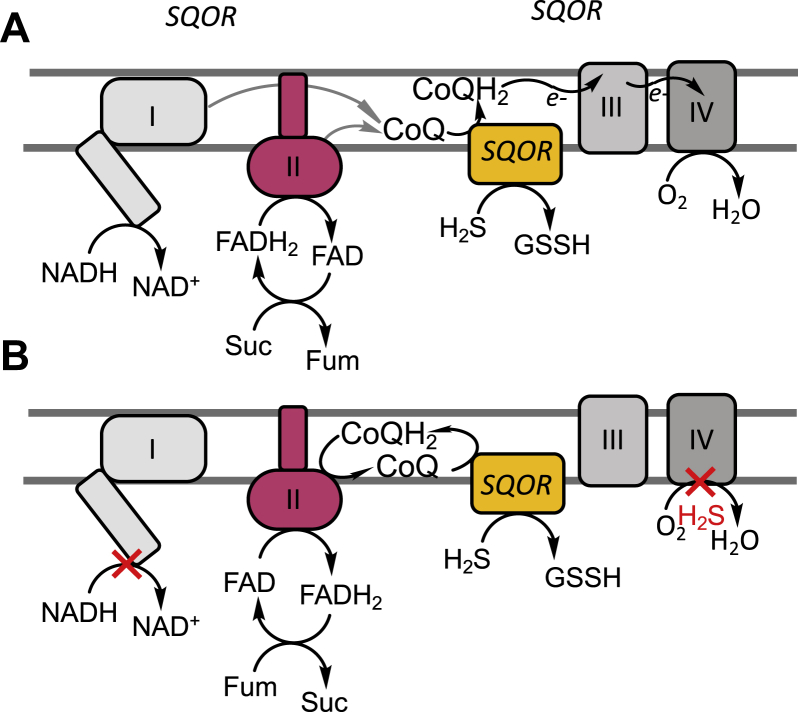
**Alternate redox cycles for disposing H**_**2**_**S.***A* and *B*, CoQH_2_ formed during H_2_S oxidation and by complexes I and II enters the ETC at the level of complex III (*A*). When complex IV is inhibited by H_2_S, blocking recycling of CoQH_2_ by complex III, CoQH_2_ can be oxidized by complex II, concomitant with fumarate reduction and succinate accumulation (*B*).

Our studies support a model for efficient H_2_S clearance by SQOR when the H_2_S concentration is low with complexes I and II competing for the CoQ pool and complex III recycling CoQH_2_ (Fig. 6*A*). However, when H_2_S concentrations rise and inhibit complex IV, utilization of fumarate as an electron acceptor by complex II sustains recycling of CoQH_2_ (Fig. 6*B*). Complex II catalyzes the reversible oxidation of succinate to fumarate (39) and exhibits similar *K*_M_ values for both substrates (40, 41). Under *in vitro* assay conditions, the ratio of succinate oxidation to fumarate reduction catalyzed by the succinate dehydrogenase component of complex II varies substantially with the electron acceptor and ranges from ∼0.1 to 50 for succinate:fumarate consumed (41). Under physiological conditions, flux through the forward *versus* reverse reaction is governed by the concentration of the respective substrates and by the potentials of the relevant redox couples. In the mitochondrial matrix (pH ∼ 7.7), the standard redox potential for the fumarate/succinate couple (E˚′ = +30 mV) is similar to that for ubiquinone/ubiquinol (+40–60 mV at pH 7.0, decreasing 60 mV per increase in pH unit (42)), but higher than of the FAD/FADH_2_ couple (−79 mV (43, 44)). The reversibility of complex II in cells is supported by its ability to sustain proficient growth on fumarate as a terminal electron acceptor when expressed under anaerobic conditions in an *Escherichia coli* strain lacking fumarate reductase (45). These data support the plausibility of complex II reversal under conditions when the ETC is blocked, and the CoQ pool is overreduced.

Modulation of H_2_S metabolism by complex I was demonstrated by its inhibition by rotenone and by NDUFS3 knockdown, both enhanced H_2_S clearance (Fig. 1, *B* and *C*), as expected, and is consistent with their increased sulfide-induced OCR compared with control cells (Fig. 3). On the other hand, SDHA knockdown decreased the efficiency of H_2_S clearance while DMF increased it (Figs. 2 and S3). Under conditions of complete coupling, for every mole of sulfide oxidized by SQOR, ETHE1 and complex IV are predicted to consume 1 and 0.5 mol of O_2_, respectively. ETHE1 is a mononuclear iron-dependent persulfide dioxygenase, which catalyzes the conversion of glutathione persulfide to sulfite (46, 47). SDHA knockdown cells exhibited increased sensitivity to H_2_S-induced inhibition of OCR and took longer to recover, while DMF reduced the time to recovery of the basal OCR (Figs. 2 and 3). Collectively, these results support our model of complex II-dependent recycling of CoQH_2_ (Fig. 6*B*). It is important to note, however, that interfering with complex II reduces but does not completely block H_2_S consumption. Thus, other mechanisms including SQOR-dependent reduction of O_2_ (Fig. S1) might contribute to H_2_S removal.

The significant decrease in thiosulfate upon silencing SDHA in murine intestinal epithelial cells (Fig. 5) is notable for three reasons. It supports the physiological relevance of reverse complex II activity for H_2_S oxidation as loss of the canonical succinate oxidation activity would be expected to stimulate SQOR-dependent H_2_S oxidation by decreasing competition for the CoQ pool. Second, the observed change in thiosulfate levels in *Sdha*^*ΔIEC*^ mice reflects the quantitatively significant impact of complex II activity in intestinal epithelial cells on systemic sulfide metabolism. Third, changes in urine and serum thiosulfate in *Sdha*^*ΔIEC*^ mice reveal the systemic impact of altered H_2_S metabolism at the host–microbe interface, which warrants further study.

We speculate that H_2_S-fueled succinate accumulation could have downstream metabolic effects. Succinate is a competitive inhibitor of α-ketoglutarate-dependent dioxygenases and its accumulation could broadly impact histone and DNA methylations (48). Furthermore, succinylation, a posttranslational modification of proteins (49), could be enhanced by H_2_S-driven succinate accumulation. Over 750 protein targets of succinylation have been identified, which are concentrated in mitochondria but also present in other compartments (50) and reversed by the NAD^+^-dependent sirtuin, Sirt5 (51). Succinylation reportedly increases complex II activity (50). We speculate that succinylation could be enhanced by the opposing effects of H_2_S on the succinate and NAD^+^ pools, in an autocorrective loop for activating complex II and prioritizing its removal.

In summary, our study reveals that metabolic reprogramming leads to the establishment of a new redox cycle between SQOR and complex II, permitting sustained H_2_S clearance. In addition to its relevance at the gut host–microbe interface, this circuitry could be important in the context of ischemia reperfusion injury. H_2_S is cytoprotective when administered at the time of reperfusion, reducing infarct size, inhibiting myocardial inflammation, and preserving mitochondrial integrity (52). The rapid reoxidation of succinate, which accumulates in the ischemic phase, drives ROS production during reperfusion (23). We posit that the cytoprotective effects of H_2_S could derive from its twin effects on complex IV inhibition and complex II reversal, thereby attenuating succinate-dependent ROS generation during reperfusion. Another cellular context in which H_2_S-mediated ETC rewiring might be relevant is during the transition from a quiescent to proliferative state. While quiescent cells primarily rely on the high energy yield of oxidative phosphorylation, proliferating cells increase aerobic glycolysis to meet their energy needs and redirect mitochondrial metabolism for macromolecular precursor synthesis (53). The potential for H_2_S to function as an endogenous modulator of energy metabolism could be significant in this context and needs to be further understood.

## Conclusions

Colonocytes are routinely exposed to H_2_S derived from microbial metabolism and are adapted to remove high concentrations of this toxic gas *via* a mitochondrial sulfide oxidation pathway that links to the electron transport chain. We have discovered that cells prioritize the removal of H_2_S when its levels are high enough to inhibit respiration, by utilizing fumarate as an alternate electron acceptor. Specifically, a new redox circuitry is established between SQOR, which reduces CoQ as it oxidizes H_2_S, and complex II, working in reverse to regenerate CoQ as it reduces fumarate. Mice with targeted deletion of complex II in intestinal epithelial cells exhibit systemic reduction in H_2_S oxidation, establishing physiological relevance of this redox circuitry and revealing a quantitatively significant contribution of colonocytes to whole-body sulfide homeostasis.

## Experimental procedures

### Materials

Sodium sulfide nonahydrate (431648), sodium sulfite (S0505), sodium selenite (S5261), CoQ (C7956), dimethyl malonate (63380), dimethyl itaconate (592498), diethyl succinate (8.00680), rotenone (R8775), dimethyl fumarate (242926), 4-chloro-7-nitrobenzofuran (163260), doxycycline (D3447), puromycin (P8833), protease inhibitor cocktail for mammalian tissue extract (P8340), RIPA lysis buffer (R0278), and apo-transferrin (T1147) were from Sigma. RPMI 1640 (11875-093), DMEM (11995-065), FBS (10437-028), trypsin-EDTA (25300-054), penicillin-streptomycin (15140-122), geneticin (10131-035), M199 (11150-059), epidermal growth factor (PHG0311), PBS (10010-023), DPBS (14040-133), and insulin (12585014) were from Gibco. Anti-Flag (20543-1-AP), anti-NDUFS3 (15066-1AP), anti-SDHA (14865-1AP), anti-GOT1 (14886-1AP), and anti-GOT2 (14800-1AP) antibodies were from Proteintech, and the secondary anti-rabbit horseradish peroxidase-linked IgG antibody (NA944V) was from GE Healthcare.

### Assays for ndSQOR-catalyzed O_2_ consumption and H_2_O_2_ production

Human SQOR was purified and embedded in nanodiscs as described previously (27). O_2_ consumption by FADH_2_ in *nd*SQOR was monitored using an O2k respirometer (Oroboros Instruments), equipped with two polarographic O_2_-sensing electrodes housed in separate 2 ml chambers. Each chamber was filled with 100 mM potassium phosphate, pH 7.4, and sulfide (100 μM) and sulfite (200 μM) were added before sealing the chambers and pre-incubating for ∼5 min at 25 °C. The reaction was initiated by injecting *nd*SQOR (100 nM) and monitored over a period of ∼10 min. Initial O_2_ concentrations were varied by aerating N_2_-purged buffer in the chambers before sealing when the desired O_2_ concentration was reached. H_2_O_2_ production was assayed using the Pierce Quantitative Peroxide Assay Kit (Thermo Fisher) according to the manufacturer’s protocol.

### Cell culture

HT29 cells were maintained in RPMI 1640 medium. HCT116, LoVo, DLD, and RKO were maintained in DMEM medium. Both RPMI and DMEM media were supplemented with 10% FBS, 100 units/ml penicillin, and 100 μg/ml streptomycin. HCEC cells were cultured as described previously (16). All cells were maintained at 37 °C with ambient O_2_ and 5% CO_2_ except HCEC, which were maintained at 2% O_2_ and 5% CO_2_.

### Ectopic expression of LbNOX and TPNOX

*Lb*NOX and mito-*Lb*NOX and pINDUCER empty vector were obtained from Addgene. The pLVX-TRE3G empty vector, TPNOX, mito-TPNOX, and pLVX TET ON were a generous gift from Dr. Valentin Cracan (Scintillon Institute). The construction of HT29 cell lines stably expressing *Lb*NOX, mito-*Lb*NOX, TPNOX, and mito-TPNOX has been described previously (19, 20). Before the start of an experiment, these cells were incubated for 24 h with 300 ng/ml doxycycline to induce *Lb*NOX expression. The cells were routinely cultured in RPMI 1640 medium supplemented with 10% FBS, 100 units/ml penicillin, 100 μg/ml streptomycin and 300 μg/ml geneticin, and 1 μg/ml puromycin.

### Generation of shRNA-mediated knockdown cells

NDUFS3 and SDHA were targeted for knockdown using shRNA purchased from MISSION shRNA Library, Sigma. The clone IDs for NDUFS3 were NM_004551.1-320s21c1 and NM_004551.1-628s21c1. The clone IDs for SDHA were NM_004168.1-619s1c1 and NM_004168.1-1643s1c1. The doxycycline-inducible GOT1 and GOT2 lentiviral constructs were subcloned into the iDox-pLKO vector as described previously (54, 55). Plasmids containing shRNA against specific genes or a scrambled sequence were submitted to the Vector Core (University of Michigan) for lentiviral packaging. For lentiviral infection, 7.5 × 10^4^ HT29 cells were seeded in a six-well plate containing 2 ml per well of RPMI 1640 medium supplemented with 10% FBS, 100 units/ml penicillin, and 100 μg/ml streptomycin. The transduction and selection protocols were the same as described for *Lb*NOX (19), and cells were selected with 1 μg/ml puromycin.

### Western blotting

TPNOX expression in HT29 cells was monitored by growing cells in a six-well plate for 24 h in RPMI 1640 medium as described above followed by a 24 h incubation with 300 ng/ml doxycycline. Then, the cells were washed with PBS twice before addition of 250 μl of RIPA lysis buffer containing 10 μl/ml protease inhibitor cocktail for mammalian tissue extracts and collected by scraping. Cells were frozen and thawed three times and centrifuged at 12,000*g* for 5 min. The protein concentration in the supernatant was measured using Bradford reagent (Bio-Rad). Protein lysates were similarly prepared from cells in which NDUFS3, SDHA, and GOT1/2 were knocked down. Following separation by 10% SDS PAGE, proteins were transferred to a PVDF membrane and incubated overnight at 4 °C with primary anti-Flag antibody at a dilution of 1:1000 for TPNOX. Antibodies against NDUFS3, anti-SDHA, GOT1, and GOT2 (14800-1AP) were used at a dilution of 1:2000. Horseradish-peroxidase-linked anti-rabbit IgG was used at a dilution of 1:10,000. Membranes were developed and visualized using the KwikQuant Digital-ECL substrate and imaging system.

### Cellular H_2_S consumption assay

Cells were grown to ∼90% confluency in 10 cm plates and on the day of experiment, washed with PBS and treated with 0.05% trypsin-EDTA (for ∼10 min at 37 °C). Then, cells were resuspended in 10 ml complete media and centrifuged for 5 min at 4 °C, 1700*g*. The cell pellet was resuspended in 1 ml modified DPBS (supplemented with 20 mM HEPES, pH 7.4, and 5 mM glucose) in a preweighed Eppendorf tube and centrifuged for 5 min at 4 °C, 1700*g*. The supernatant was discarded, and the pellet weight was determined. Cells were suspended in modified DPBS to make a 5% cell suspension (w/v) in a 1 ml Eppendorf tube. When the effects of dimethyl malonate (DMM,10 mM) or dimethyl itaconate (DMI, 0.25 mM) were tested, cells were preincubated for 3 h with each reagent before making a 5% cell suspension in which the same concentrations of DMM and DMI were included followed by addition of 100 μM Na_2_S. Alternatively, when dimethyl fumarate (DMF, 100 μM) and diethyl succinate (DES, 5 mM) were tested, these reagents were added to a 5% cell suspension in modified DPBS for 5 min prior to addition of 100 μM Na_2_S. The suspension cultures were incubated at 37 °C with shaking (75 rpm). Samples (45 μl) were collected at time 0 and 10 min, mixed with 1 M Tris base (2.5 μl), and stored in dry ice. Control samples containing 10 mM DMM, 0.25 mM DMI, 100 μM DMF, or 5 mM DES and 100 μM Na_2_S in modified DPBS were incubated in parallel, and the concentration of H_2_S lost from these samples was subtracted from the values obtained from the cell suspension samples containing the same reagents.

### Monobromobimane derivatization of sulfide and HPLC analysis

The samples from the H_2_S consumption assay described above were thawed and mixed with 2.5 μl of 60 mM monobromobimane (in DMSO) and incubated in the dark at room temperature for 10 min followed by addition of 100 μl of metaphosphoric acid solution (16.8 mg/ml). The samples were vortexed and centrifuged for 5 min at 4 °C and 10,000*g*. The supernatant was collected in the dark and stored at −20 °C until further use. The samples were analyzed using a Zorbax Eclipse XDB-C18 column (5 μm, 4.6 × 150 mm, Agilent) as described previously (8). Peaks were detected using excitation at 390 nm and fluorescence emission at 490 nm. A calibration curve with known concentrations of sodium sulfide was used to determine the concentration of H_2_S in samples.

### Metabolomics analysis

Metabolomics analysis on HT29 cells treated ±100 μM Na_2_S for 1 h was performed as described previously (16).

### OCR measurements

Oxygen consumption was measured using the O2k respirometer. Cells were grown to ∼90% confluency in 10 cm plates and on the day of experiment, washed with PBS, and then trypsinized with 1.5 ml of 0.05% trypsin-EDTA for ∼10 min at 37 °C. Then, the cells were resuspended in 10 ml of complete medium and centrifuged for 5 min at 1700*g*, 4 °C. The cell pellet was resuspended in 1 ml of modified DPBS in a preweighed Eppendorf tube, the suspension was centrifuged for 5 min at 1700*g*, and the weight of the pellet was recorded. The cells were suspended in modified DPBS to make a 5% cell suspension (w/v), which was stored on ice. At the start of the experiment, the cell suspension was diluted to 1% or 1.5% (for NDUFS3 knockdowns which showed lower basal OCR). The cell suspension was placed in the respirometer chamber and the OCR was allowed to stabilize over ∼15 to 20 min at 37 °C with constant stirring at 750 rpm. Na_2_S (from a freshly prepared 10 mM stock solution in water) was injected into the sample to give the desired final concentration (10–30 μM) per injection.

### Mice

*B6.Cg-Tg(Vil-cre)1000Gum/J* mice were purchased from the Jackson Laboratory. C57BL/6N-*Sdha*^*tm2a(KOMP)Wtsi*^ mice were obtained from the Knock Out Mouse Project (KOMP) repository, University of California, Davis and bred to *ACTFLPe* mice to excise the FRT-flanked region. The resulting *Sdha*^*fl/fl*^ mice were bred to *Vil1-Cre* mice to create *Vil1-Cre Sdha*^*fl/fl*^ (*Sdha*^Δ/IEC^) mice (32). Then, 12 to 15 week-old mice were used in our experiments. The mice were maintained under specific pathogen-free conditions following procedures approved by the University of Michigan Committee on the Use and Care of Animals, which are based on the University of Michigan Laboratory Animal Medicine guidelines.

### Statistical analysis

Statistical analyses were performed using GraphPad Prism 9. Two-tailed tests were used for all t-tests. Errors on measurements are represented as standard deviation.

## Data availability

All data are contained within the manuscript and in the supplemental section.

## Supporting information

This article contains supporting information.

## Conflict of interest

C. A. L. is a consultant for Astellas Pharmaceuticals and is an inventor on patents pertaining to Kras regulated metabolic pathways, redox control pathways in pancreatic cancer, and targeting the GOT1-pathway as a therapeutic approach.
