# Supplementary material for: A redox cycle with complex II prioritizes sulfide quinone oxidoreductase-dependent H2S oxidation
Source: J Biol Chem. 2021 Nov 19;298(1):101435. doi: 10.1016/j.jbc.2021.101435 (PMC8683732; doi:10.1016/j.jbc.2021.101435)
Supplement: Supplemental Figures S1–S10 [file mmc1.docx]

Supporting Information

A redox cycle with complex II prioritizes sulfide quinone oxidoreductase dependent H_2_S oxidation

Roshan Kumar,^1^ Aaron P. Landry,^1^ Arkajit Guha,^1^ Victor Vitvitsky,^1^ Ho Joon Lee,^2^ Keisuke Seike,^3^ Pavan Reddy,^3^ Costas A. Lyssiotis^2^ and Ruma Banerjee^•^

Departments of ^1^Biological Chemistry, ^2^Molecular and Integrative Physiology, and ^3^Internal Medicine, Michigan Medicine, University of Michigan, Ann Arbor, MI 48109

***Short Title***: H_2_S induces complex II reversal

^•^Corresponding author: email: rbanerje@umich.edu

**This file includes:**

Figs. S1 – S10


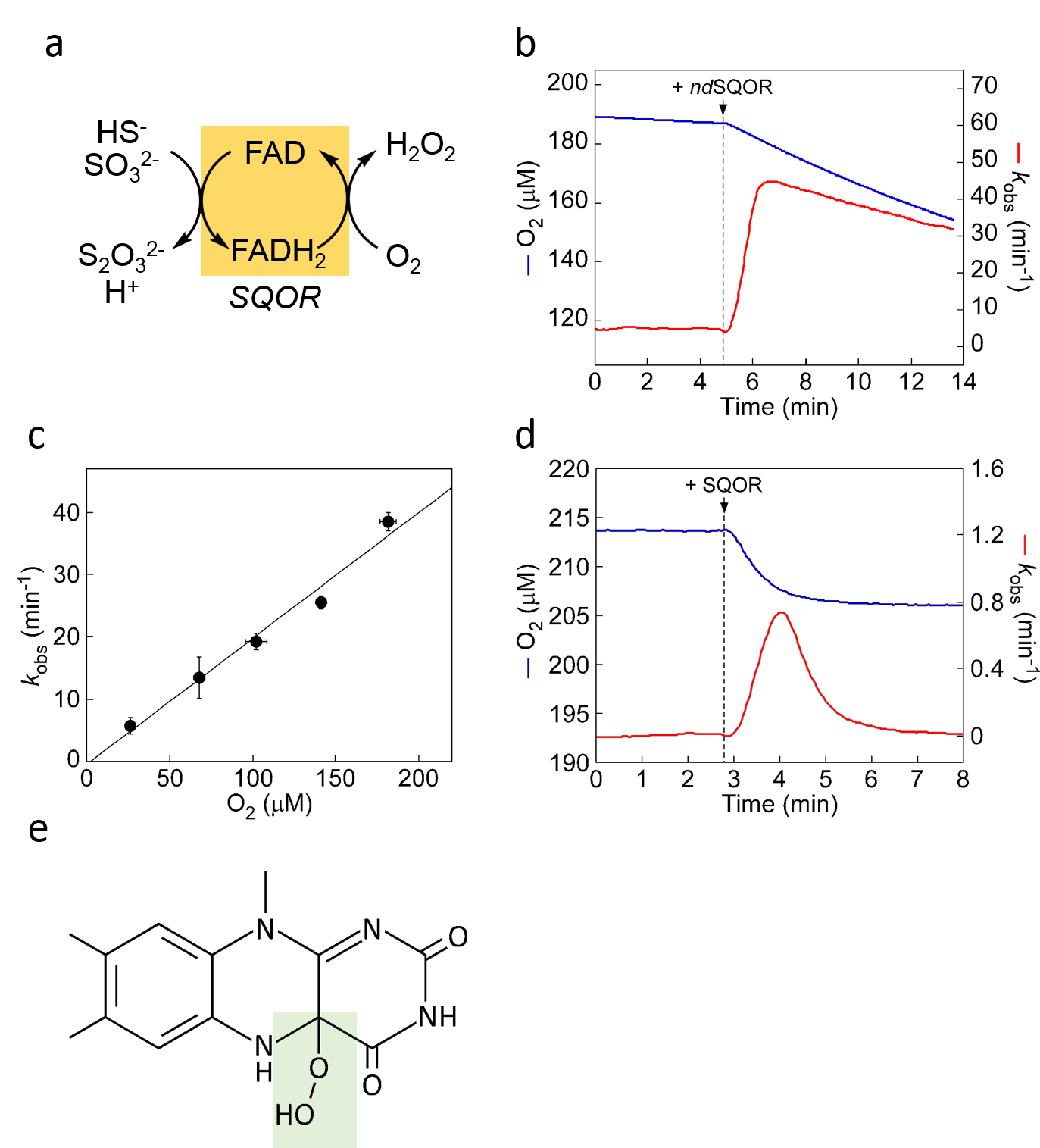


**Figure S1. Kinetics of O_2_ consumption catalyzed by *nd*SQOR.** **(a)** Scheme showing SQOR-catalyzed H_2_O_2_ production. **(b)** Oxygen consumption kinetics in the presence of *nd*SQOR (100 nM) added to a reaction mixture containing sulfide (100 μM) and sulfite (200 μM) in 100 mM potassium phosphate buffer, pH 7.4. **(c)** Dependence of the *nd*SQOR-catalyzed O_2_ consumption rate on O_2_ concentration. **(d)** Oxygen consumption kinetics in the presence of SQOR (7.5 μM) added to a reaction mixture containing sulfide (10 μM) and sulfite (15 μM) in 100 mM potassium phosphate buffer, pH 7.4. **(e)** Structure of the proposed 4a-hydroperoxy FAD intermediate. The data are representative (b and d) or the mean ± S.D. (c) of three independent experiments.

**
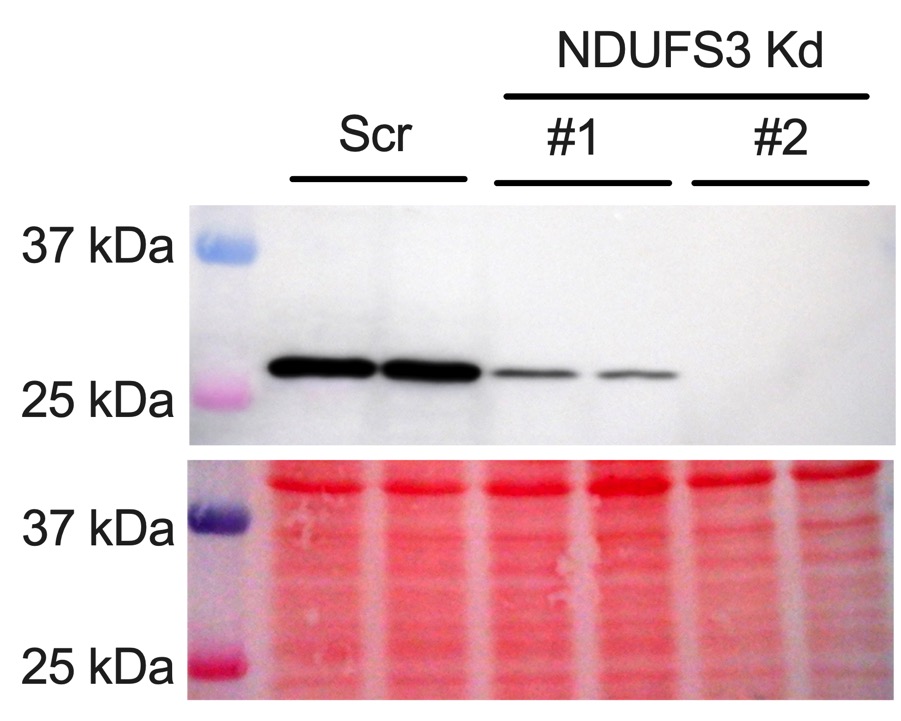
**

**Figure S2. Validation of NDUFS3 knockdown.** Western blot validation of NDUFS3 knockdown with two targeting sequences (#1 and 2, each loaded in duplicate) in HT29 cells. The loading controls (*lower* *panel*) represents total protein detected with Ponceau S red stain.

**Figure S3. Fumarate acts as terminal electron acceptor in colon cancer cells.** DMF (100 μM) increased H_2_S oxidation in **(a)** HCT116, **(b)** LoVo, and **(c)** DLD cells but not in (**d**) RKO cells. The data are the mean ± S.D. of 3-5 independent experiments.

**Figure S4. Complex II activity influences H_2_S clearance by HT29 cells.** Complex II inhibitors dimethyl malonate (DMM, 10 mM) and dimethyl itaconate (DMI, 0.25 mM) decreased H_2_S consumption, whereas diethyl succinate (DES 5 mM) did not show a significant effect. The data represent the S.D. of 3 independent experiments. The control data shown here is the same as for the experiment in Fig. 1b, which was performed in parallel.

**Figure S5. Validation of SDHA knockdown in HT29 cells using two shRNA targeting sequences.** Western blot analysis (*top*) and Ponceau S red staining (*bottom*) show expression SDHA in HT29 cells transfected with scrambled or two SDHA targeting sequences (#1 and 2).

**
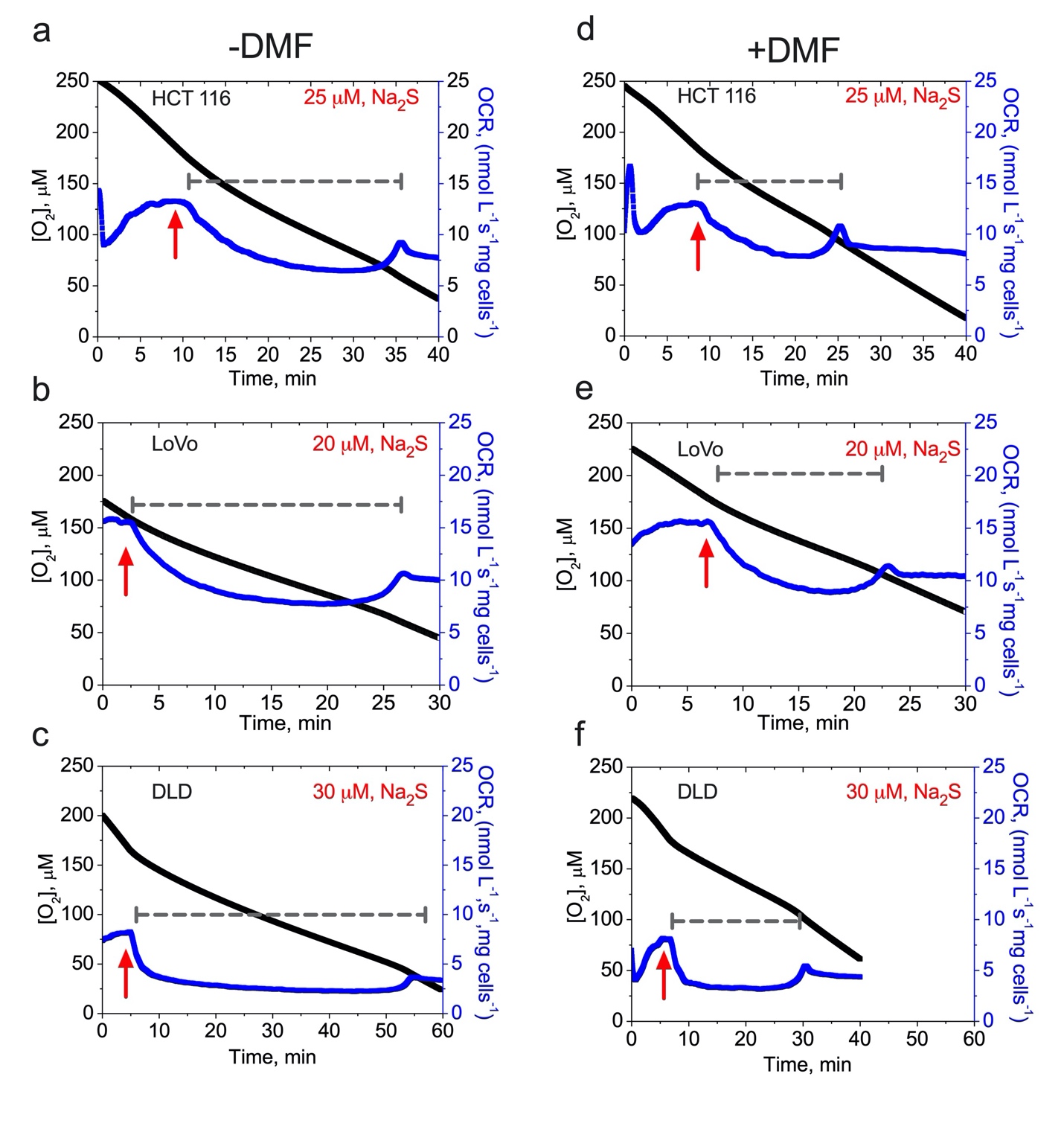
**

**Figure S6. DMF shortens recovery to the basal respiration rate following inhibition by H_2_S.** Comparison of recovery time to basal OCR following Na_2_S exposure in the absence (**a,b,c**) and presence (**d,e,f**) of DMF (200 µM) in three colorectal cancer cell lines (HCT116, LoVo and DLD). The red arrows indicate when Na_2_S was added. The length of the gray dashed lines represent the recovery time. The data are representative of 3 independent experiments.

**Figure S7. DMF does not affect recovery time to basal respiration rate in SDHA knockdown cells treated with H_2_S.** Comparison of the time to recovery of basal OCR following H_2_S (30 μM) treatment in the absence (**a**) and presence (**b**) of DMF (200 µM) in SDHA knockdown HT29 cells. The red arrows indicate when Na_2_S was added. The length of the gray dashed lines represents the recovery time. The data are representative of 3 independent experiments.

**Figure S8. Complex I and II influence H_2_S-responsive OCR.** Comparison of OCR activation by H_2_S (10 μM) in scrambled **(a)**, SDHA knockdown **(b)**, and NDUFS3 knockdown **(c)** HT29 cells. The traces are representative of 3-5 independent experiments. The red arrows indicate when Na_2_S was added.

**Figure S9. Validation of GOT1 and GOT2 knockdown in HT29 cells. (a)** Expression of GOT1 (*upper*) and GOT2 (*middle*) in GOT1 knockdown cells. **(b)** Expression of GOT2 (*upper*) and GOT1 (*middle*) in GOT2 knockdown cells. Equal loading as indicated by Ponceau S red staining, is shown in the *lower* row in both panels.


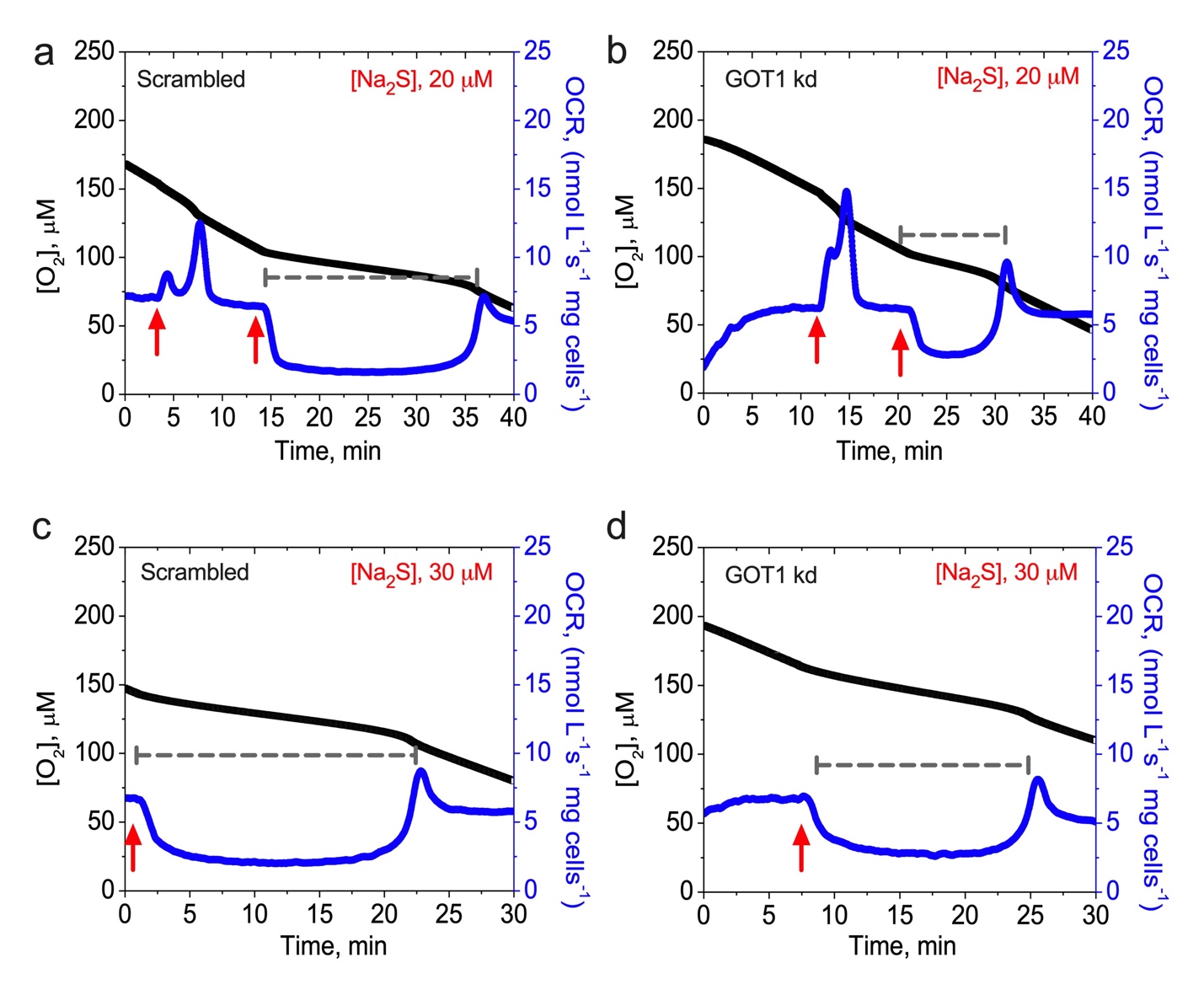


**Figure S10. GOT1 knockdown promotes H_2_S clearance.** Comparison of the time to recovery of basal OCR following a second injection of Na_2_S (20 μM) to (**a**) scrambled versus (**b**) GOT1 knockdown HT29 cells. A similar comparison following injection of a higher concentration of Na_2_S (30 μM) to (**c**) scrambled, versus (**d**) GOT1 knockdown cells. The red arrows indicate when Na_2_S was added. The length of the gray dashed lines represents the recovery time. The data are representative of 3 independent experiments.
